# Functional Status Correlates of Change and Stability in Appraisal after Spine Surgery: Earlier versus Later Effects

**DOI:** 10.3390/jpm14030329

**Published:** 2024-03-21

**Authors:** Carolyn E. Schwartz, Katrina Borowiec, Bruce D. Rapkin, Joel A. Finkelstein

**Affiliations:** 1 DeltaQuest Foundation, Inc., Concord, MA 01742, USA; katrina.borowiec@bc.edu; 2 Departments of Medicine and Orthopaedic Surgery, Tufts University School of Medicine, Boston, MA 02111, USA; 3 Department of Measurement, Evaluation, Statistics, & Assessment, Boston College Lynch School of Education and Human Development, Chestnut Hill, MA 02467, USA; 4 Department of Epidemiology & Population Health, Albert Einstein College of Medicine, Bronx, NY 10461, USA; bruce.rapkin@einsteinmed.org; 5 Division of Spine Surgery, Sunnybrook Health Sciences Centre, Toronto, ON M4N 3M5, Canada; joel.finkelstein@sunnybrook.ca; 6 Department of Surgery, University of Toronto, Toronto, ON M4N 3M5, Canada; 7 Division of Orthopedic Surgery, Sunnybrook Health Sciences Centre, Toronto, ON M4N 3M5, Canada

**Keywords:** cognitive appraisal, change, stability, disability, spine surgery

## Abstract

Spine surgery generally yields a notable improvement in patients’ health state, and there is variability in measured patient outcomes after spine surgery. The present work aimed to describe for clinicians how appraisal underlies their patients’ experience of healthcare interventions. This prospective longitudinal cohort study (n = 156) included adults undergoing spine surgery for degenerative spinal conditions. The analysis was a descriptive illustration of the relationship between change in the spine-related disability using the Oswestry Disability Index and change in cognitive-appraisal processes using the Quality-of-Life Appraisal Profilev2-Short Form, early versus later during the recovery trajectory (i.e., between baseline and 3 months post-surgery; and between 3 and 12 months post-surgery). Cognitive-appraisal processes related to Sampling of Experience showed greater change soon after surgery, whereas Standards of Comparison appraisals changed more later in the recovery trajectory. Different appraisal processes were emphasized by patients who reported worsening of the spine-related disability, as compared to those who reported no change or improvement. These findings suggest that changes in appraisal differ depending on the individual’s experience of the impact of spine surgery. Appraisal processes thus reflect an ongoing dynamic in adaptation to changing function.

## 1. Introduction

Spine surgery generally yields a notable improvement in patients’ health state, reducing pain and increasing function [1,2,3]. There is, however, some variability in measured patient outcomes after spine surgery, related to demographic [4], clinical [5], psychological [6,7,8], and other contextual factors [9]. One notable factor of particular relevance to personalized medicine is cognitive-appraisal processes (i.e., how people recall past experiences and to whom they compare themselves) [10]. Past research has documented that appraisal processes are relevant to chronic pain [11,12,13,14] and spine-specific disability and mental-health functioning and suggested that such processes are modifiable targets of intervention [15].

The concept of appraisal has a long history in psychological research. Perhaps the first mention of appraisal was in the Folkman and Lazarus stress and coping model [16], in which the term was used to refer to whether an individual thought of a stressor as controllable by doing something constructive (i.e., problem-focused coping), or as having to be accepted (emotion-focused coping) [16]. In the field of quality-of-life (QOL) research, appraisal processes focused more on how individuals thought about health when answering survey questions [17,18]. These cognitive-appraisal processes were characterized as comprising four domains: Frame of Reference, Sampling of Experience, Standards of Comparison, and Combinatory Algorithm (i.e., patterns of emphasis) [18]. Such processes were explicitly considered in the Rapkin and Schwartz Appraisal Theory [18], which expanded upon the Sprangers and Schwartz Response Shift Theory [19]. Both theoretical models sought to explain adaptation effects in the face of changing health (i.e., a “catalyst”), and both included reference to stable characteristics of the individual (i.e., “antecedents”), behavioral “mechanisms” for reacting to these health-state changes (i.e., “mechanisms”), response-shift effects, and an unexpected QOL outcome (i.e., higher or lower QOL than would be expected). Response shift was conceptualized as a change in the meaning of one’s self-evaluation of a target construct as a result of: (a) a change in the respondent’s internal standards of measurement (scale recalibration, in psychometric terms); (b) a change in the respondent’s values (i.e., the importance of component domains constituting the target construct); or (c) a redefinition of the target construct (i.e., reconceptualization) [18,19]. Whereas the earlier model provided examples of response shift that overlapped considerably with “mechanisms” (e.g., goal reordering as a both change in priorities/values and a coping mechanism), the Rapkin and Schwartz model distinguished cognitive-appraisal processes as a part of the model, resulting from both antecedents and mechanisms. This model operationalized response shift as “when change in appraisal explained the discrepancy between expected and observed QOL [18]”. Thus began a long research path developing and validating a series of increasingly viable measures of appraisal [18,20].

In this long research path, cognitive-appraisal processes were documented to explain substantial variance in a wide range of patient samples [21], and to help to explain why two individuals in identical health states rate their QOL differently [22]. Appraisal assessment helps to identify and explain how contextual and psychological factors matter in patients’ subjective evaluation of their physical and emotional health [21,22,23]. With this growth in the evidence base for appraisal, there has also been increasing parsimony in appraisal assessment [20] and in statistical methods for working with appraisal [23]. These statistical methods have relied on data reduction and relatively complex multivariate analyses which, though useful for summarizing findings at the aggregate level, may also make the findings hard to parse for clinicians and others not familiar with complex statistical modeling.

The present work was motivated by the gap between the abovementioned complexity and the need for clinicians to understand how appraisal underlies their patients’ experience of healthcare interventions. Focusing on the experience of spine surgery and subsequent recovery over time (catalyst), we sought to describe the ramifications of the surgery for patients along the improvement continuum in the initial three months after surgery, and in the subsequent nine months post-surgery. Two research questions were asked: (1) What drives changes in appraisal after spine surgery? (2) How does change in appraisal vary in response to change in functioning early versus later post-surgery?

## 2. Materials and Methods

### 2.1. Sample and Design

This prospective longitudinal cohort study included adults recruited from a spine-surgery practice at a Canadian academic teaching hospital. Eligibility criteria included being over the age of 18 and having undergone elective spinal decompression and/or fusion surgery for diagnoses of disc herniation, radiculopathy/sciatica, spinal stenosis with neurogenic claudication, or degenerative spondylolisthesis. Exclusionary criteria entailed having had prior lumbar surgery at the same level, or being unable to understand and complete the English survey-related documents. All patients provided written informed consent prior to study entry. Data were collected online or by mail pre-surgery and at approximately 3 and 12 months post-surgery using a secure, Health Information Portability and Accountability Act (HIPAA)-compliant interface [24]. The study was reviewed and approved by the Sunnybrook Health Centre Research Ethics Board (#2591).

### 2.2. Measures

Spine-specific disability was measured using the Oswestry Disability Index (ODI) [25]. This measure is the most commonly used tool in both operative and non-operative spine-patient cohorts. The ODI assesses the level of pain and interference with a range of activities of daily living and physical activities. Each item is scored from 0 to 5 (0, severe disability, to 5, which is no disability). The ODI is scored such that higher scores reflect more disability, with a range from 0 to 100.

Cognitive-appraisal processes were measured using items from two domains of the QOL Appraisal Profile_v2_ Short-Form (QOLAP_v2_-SF) [20]. The 14 *Sampling-of-Experience* items query what types of experiences people recall or think about when responding to QOL measures. The 8 *Standards-of-Comparison* items query to whom or what the individual compares themself to when thinking about QOL. Items utilized a 5-point rating scale (Never, Rarely, Sometimes, Often, Always), with higher values reflecting more endorsement. “Not applicable” responses were recoded to “Never” for the analysis.

To describe the sample, demographic and clinical characteristics were collected, including age, gender, smoking status, and education. Clinical data included diagnosis, primary procedure, number of vertebrae fused if fusion surgery had occurred, pain-medicine frequency, and comorbidities, the latter of which was assessed using the Self-Administered Comorbidity Questionnaire [26].

### 2.3. Statistical Analysis

This analysis is a descriptive illustration of the relationship between change in spine-related disability and change in cognitive-appraisal processes. We conducted dependent samples’ t-tests within each group during two time intervals: between baseline and 3 months post-surgery (*1st interval*); and between 3 and 12 months post-surgery (*2nd interval*). Descriptive statistics summarized the mean change of these ODI scores and cognitive-appraisal items for each time interval.

The study sample was sorted into groups according to the magnitude of change in ODI scores using Cohen’s *d* in each of the two time intervals according to the following distribution-based criteria [27]: no effect-size (ES) change = *d* < 0.2; small ES change = *d* ≥ 0.2 and <0.49; medium ES change = *d* ≥ 0.5 and <0.79; and large ES change = *d* ≥ 0.8. Cohen’s *d* was also used to characterize the magnitude of change in cognitive-appraisal processes. Additionally, since Hedge’s *g* is considered by some to be more accurate or conservative for smaller sample sizes because the mean difference is divided by the sample variance rather than the within-sample standard deviation (SD) in the denominator [28,29,30], we also considered results using Hedge’s *g*^1^ for the appraisal comparisons. *Stability* in cognitive-appraisal processes was defined as a mean change in the QOLAP_v2_-SF item response of less than a small ES (i.e., *d* < 0.2 SD of the baseline mean), and *change* was defined as a change of at least 0.2 SD of the baseline mean. An analysis of variance (ANOVA) tested group differences in QOLAP_v2_-SF item response at each time interval.

#### Software

Data were analyzed using IBM SPSS version 29 [31] and Microsoft Excel 365.

## 3. Results

Table 1 provides a summary of baseline demographic and clinical characteristics of the whole study sample. The study sample included 156 people who underwent spine surgery. Most (63%) patients received a laminectomy/discectomy; 12% received instrumentation/fusion; and 19% instrumentation/fusion and laminectomy/discectomy.

### 3.1. Change-Group Differences in ODI

The study sample had an average ODI score of 46.7, 23.1, and 22.0 at baseline (pre-surgery), and 3 and 12 months post-surgery, respectively. The average change was −23.6 points between baseline and 3 months post-surgery and was −1.1 points between 3 and 12 months post-surgery. Based on the present sample’s standard deviation of 16 on the baseline ODI, a change score of 3.2 points represented a small ES change, of 8 points a medium ES, and of 12.8 points a large ES.

Based on the ODI change scores in the baseline-to-3-month and 3-to-12-month windows, the sample was stratified into five groups for each time interval: (1) Disability worsened; (2) No effect; (3) Small effect; (4) Medium effect; and (5) Large effect. Table 2 displays the crosstab comparing group membership in the two time intervals.

Nine patients of the total sample of one hundred fifty-six worsened in the first interval (i.e., baseline to 3 mo.). Among these 9 patients, 1 continued to worsen, 2 had a small ODI improvement, and 6 had a large ODI improvement at the second interval. Among the 10 who had no change in the first interval, in the second interval 6 worsened, 1 continued to have no change, and 1 and 2 had small and medium ODI improvements, respectively. Among the 14 with small changes in the first interval, 4 worsened, 4 had no change, and 1, 1, and 4 had small, medium, and large ODI improvements, respectively, at the second interval. Among the 17 with a medium change in the first interval, 6 worsened, 1 had no change, and 2, 3, and 5 had small, medium, and large ODI improvements, respectively, during the second interval. Among the 106 with large improvements in the first interval, by the second interval, 47 worsened, 17 had no change, and 18, 9, and 15 had small, medium and large ODI improvements, respectively.

**Figure 1:**
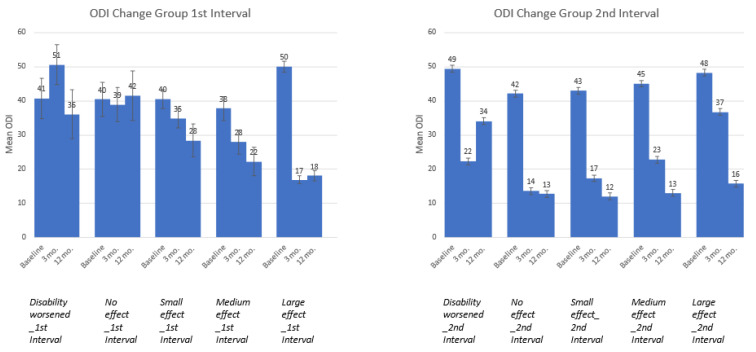
ODI means and standard errors at baseline, 3 mo. and 12 mo. by ODI-change groups.

A comparison of ODI trajectories by time interval is shown in Figure 1. In total, the figure displays 10 groups, since there are five groups per interval. Overall, the five groups derived from the first interval data differed on ODI at baseline (F = 3.962, df = 4, *p* = 0.004), and a visual inspection of Figure 1 suggests that the large ES group had the worst ODI scores (i.e., highest scores) at baseline. Post-hoc Scheffe comparisons suggested a trend (*p* = 0.06) difference between the medium and large ES groups at the first interval, but the small samples sizes for all but the large ES group undermined our confidence in the statistical power of this analysis. In contrast, the groups derived using second-interval ODI scores did not differ from each other at baseline but did differ at 3 and 12 months post-surgery (F = 1.37, 9.52, and 16. 88; *p* = 0.247, 0.0001, and 0.0001, respectively). Of note, among the second-interval group whose disability worsened, their ODI did not revert to their baseline score. Furthermore, on average, this group’s ODI change still represents a large effect size improvement at 12 months post-surgery relative to baseline. The other groups showed continued ODI-score improvement, with the second-interval no-effect group showing maintenance of a large effect immediately after surgery, and the other groups showed more of a step-function improvement trajectory.

### 3.2. Appraisal Change over Time

**Figure 2:**
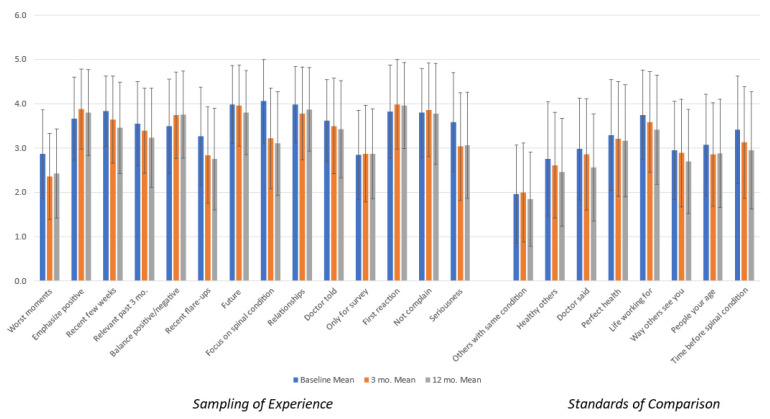
Overall appraisal means and standard deviations by time point.

Figure 2 shows the appraisal means for the overall sample, revealing that over time patients generally de-emphasized their worst moments, recent flare-ups, and their spinal condition. The error bars shown on the figure suggest that there is substantial variability at each time point, which is consistent with the premise of the appraisal theory that notable differences in appraisal processes appear both between individuals and within individuals over time in response to a catalyst (i.e., health-state change) [18,21,22].

Accordingly, Table 3 provides a comparison of changes in appraisal item endorsement over time by the ODI-change group for the two time intervals using Cohen’s *d* ES. (Due to concerns about the small sample sizes in some groups, we also evaluated Hedge’s g in the two time intervals and found highly similar results). Conditional formatting indicates the magnitude and direction of the ESs: the more saturated the color, the larger the effect, and pink fill indicates a negative direction (i.e., later appraisal-endorsement items are smaller than earlier scores) while green fill indicates a positive direction (i.e., later appraisal-endorsement items are larger than earlier scores).

During the first interval, individuals decreasingly tended to focus on their spinal condition when considering people in disability-worsened group as compared to the large-effect (improvement) group. In fact, focusing on one’s spinal condition had the largest magnitude of Cohen’s *d* value for the large-effect group. Additionally, while the disability-worsened group increased their focus on the worst moments and the seriousness of their condition during the first interval, the large-effect group decreased their focus on these areas to a similar degree. Finally, during the first interval, those in the medium and large ES groups did not shift in their focus on what their doctor told them, whereas there was a small increase for those whose disability worsened and a small decrease for those in the no-effect or small-effect groups.

During the second interval, while individuals whose disability worsened tended to increasingly focus on their spinal condition, those whose disability improved decreased their focus regarding this appraisal process. Further, whereas those whose disability worsened or did not change decreasingly focused on balancing the positive and negatives, those whose disability improved increasingly focused on this appraisal process. Next, whereas most comparisons of oneself to other standards decreased across all groups, for those whose disability improved, only a small number of individuals increased their comparison with perfect health. Finally, the medium-effect group increased their focus on balancing the positives and the negatives, while decreasing their focus on others with the same spinal condition. This pattern was not observed for those in the large-effect group.

In addition to group differences within each interval, there were also notable patterns of differences between the first and second interval. For example, there were more medium and large changes in the first as compared to the second time interval. Further, there were notable group differences in the magnitude and direction of appraisal change in the first interval, which are seen to a lesser degree in the second interval. For example, individuals whose disability worsened tended to increase their endorsement of selected appraisal processes (e.g., worse moments, the seriousness of their condition, comparing themselves to the life they are working for, etc.), whereas those with no disability change (i.e., the no-effect group) tended to decrease their endorsement of other appraisal processes (e.g., emphasizing the positive, focusing on the future, their spinal condition, or relationships, and comparing themselves to healthy others). Further, those with small, medium or large ES changes in ODI in this first interval tended to increase their focus on still other appraisal processes, increasingly focusing on emphasizing the positive, balancing the positives and the negatives, and sharing their first reaction, and decreasingly focus on their worst moments. In the second time interval, the effect sizes were generally small, and fewer group differences emerged in the direction of appraisal change.

**Figure 3:**
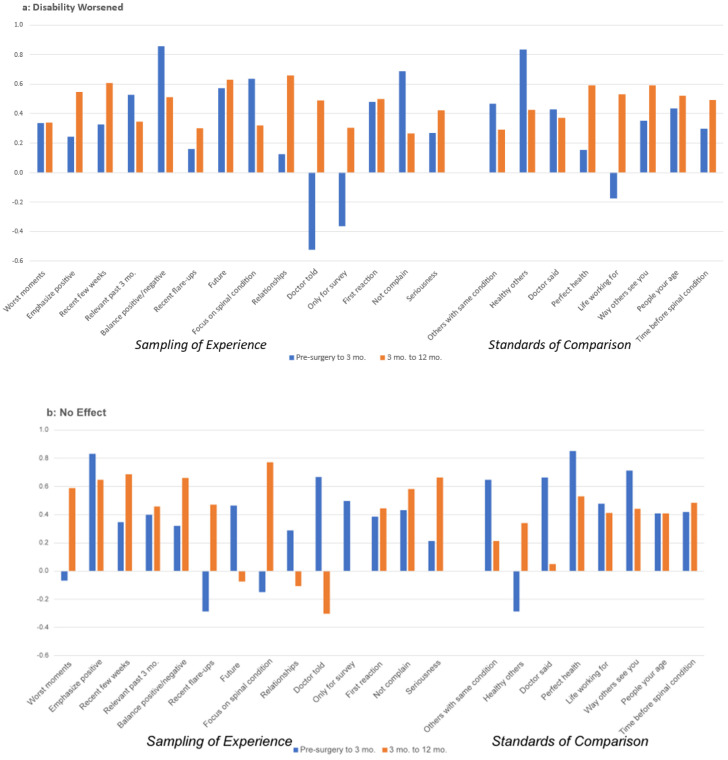

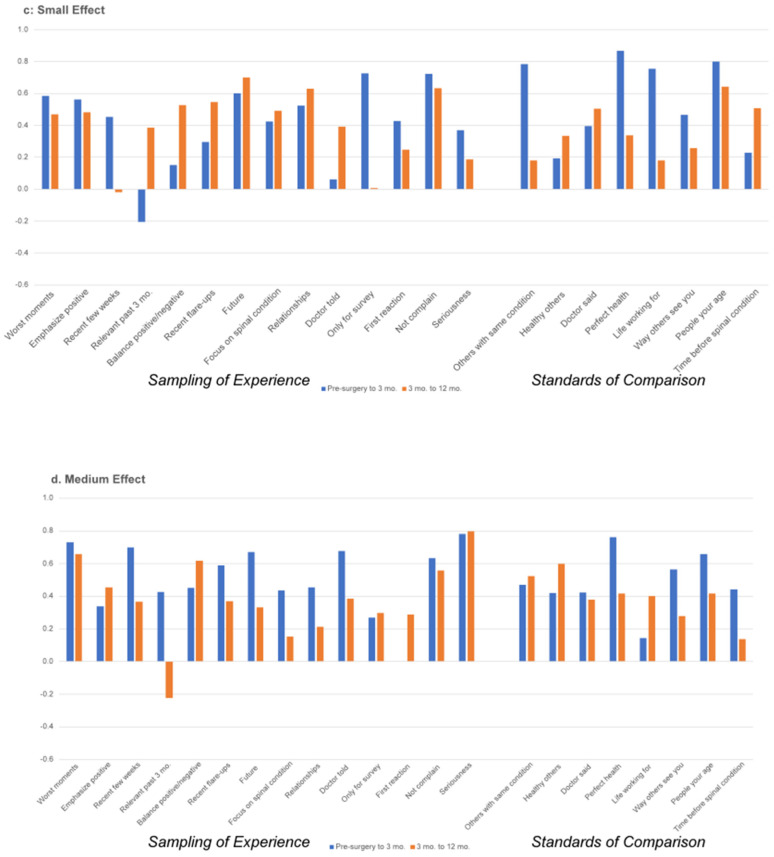

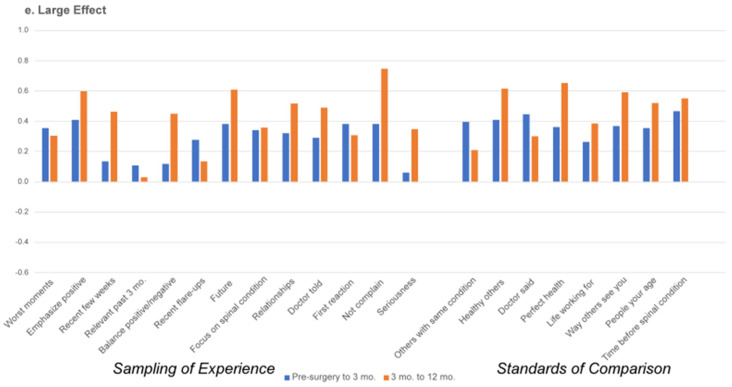
(**a**–**e**) Appraisal item stability by magnitude of ODI change: comparison between first and second time intervals.

Figure 3a–e display the Pearson correlation coefficients between appraisal processes in the first and second interval by ODI-change group. These figures suggest that for those with large ES improvements in spine-related disability, there is increasing stability (i.e., a higher correlation) in appraisal processes used by those large-ES patients in the second interval as compared to the large-ES patients in the first interval post-surgery. In other words, among patients who experienced a large reduction in spine-specific disability between 3 and 12 months post-surgery, there was consistent endorsement of specific appraisal processes (Figure 3e). By comparison, there was lesser consistency in appraisal processes endorsed in those patients who experienced a large reduction in spine-specific disability between pre-surgery and 3 months post-surgery (Figure 3e). For those with small and medium ES improvements, there are less apparent differences in the stability of appraisal processes, and the earlier interval often has higher correlations between pre-surgery and 3 months).

## 4. Discussion

This study is among the first to address the stability or changeability of QOL appraisal processes in the context of recovery from a major health intervention. We found that proximity to the catalyst of spine surgery led to more changes in Sampling of Experience appraisals (i.e., more changes in appraisal processes in the first interval, not the second interval), which is consistent with the response-shift theory [18]. In contrast, we found that changes in Standards of Comparison appraisals were more prominent *later* in the recovery trajectory rather than earlier. Further, the ODI-change groups were more similar in their changes in Standards of Comparison in this second interval, with almost all changes suggesting decreasing endorsements of such comparisons. Finally, we found that those whose disability decreased quite a bit in the second time interval exhibited greater stability overall in the appraisal processes endorsed. In contrast, the other disability-change groups showed more changeability in appraisal within and across time intervals.

Our results also underscore the personalized nature of ODI change after surgery. Given that spinal disorders are degenerative processes, it is likely that some deterioration over an extended time period after surgery is due to the natural history of continued progression. In some cases, individuals may not have shown improvement at three months, but then show considerable improvement at twelve months post-surgery. Further, al-though not directly related to the research question at hand, our findings also have implications for the concept of a minimal clinically important difference. The ODI change that reflected a large ES change using distribution-based methods (i.e., based on the standard deviation) was also the same number as that suggested by Copay and colleagues as a *minimal* clinically important difference (emphasis added) [32] and smaller than the number suggested by Nakarai and colleagues [33]. Recent systematic reviews also noted a wide range of MCID values for the same patient-reported spine-outcome measure [34,35], underscoring why it is problematic to treat it as a stable and consistent indicator of minimally important change. In addition to this empirical evidence of the variability and sample-specific nature of the MCID, the response-shift theory would hypothesize that the magnitude of the MCID would depend on many contextual factors, including stable characteristics of the individual (i.e., antecedents), the individual’s coping approaches (i.e., mechanisms), the cognitive-appraisal processes favored by the individual at a particular time (i.e., appraisal), and the impact of the catalyst on perceived functional change (i.e., change in QOL).

### 4.1. Clinical Implications

Our findings suggest that changes in appraisal differ depending on the individual’s experience of the impact of spine surgery. Building on a substantial evidence base documenting the effectiveness of cognitive–behavioral treatments for chronic pain [36], past research on appraisal has noted that some appraisal processes, such as focusing on the positive, appear to be adaptive because they co-vary with better outcomes [37,38]. The present work seems to provide a more nuanced perspective, suggesting that when patients’ outcomes are better, they focus on more positive appraisal processes. Conversely, when their outcomes are worse, they focus on more negative appraisal processes. Thus, actual functioning and self-reported outcomes are always a product of an ongoing “dialogue” between the perception and interpretation of one’s level of and change in functioning. This is akin to the chicken-and-egg question: are positive appraisals supportive of better outcomes or are better outcomes causally related to more positive appraisals? Future research might formally test this research question by randomizing patients to a coaching intervention where more positively focused appraisals were emphasized and comparing the impact of the intervention as a function of improvement in spine-related disability.

### 4.2. Limitations

The present work is limited by the relatively small ODI-change subgroup sample sizes for all but the large-ES group in the first interval and the disability-worsened group in the second interval. Although we used ES rather than p-values to characterize notable changes, the study findings must be interpreted with caution and should be replicated in more robust sample sizes. Future work in larger patient samples might stratify the analyses by age group (e.g., young adult vs. older adult) and by diagnosis to investigate whether findings differ by group. Additionally, future work might also consider the impact of psychological profiles as moderators of the appraisal–outcome relationship.

## 5. Conclusions

Cognitive-appraisal processes related to Sampling of Experience showed greater change soon after surgery, whereas Standards of Comparison appraisals changed more later in the recovery trajectory. Among the small proportion of patients who experienced a worsening of spine-related disability, they tended to focus on and emphasize different appraisal processes than those who experienced no change. Among the larger proportion of patients who experienced greater degrees of improvement, they tended to emphasize still other appraisal processes. These findings suggest that appraisal processes reflect an ongoing dynamic in adaptation to changing function.

## Figures and Tables

**Table 1:** Descriptive statistics of study sample †.

	Overall (N = 156)	
Variable	Mean	SD
**Age (at time of surgery)**	60.9	14.8
Range	20–87	
Missing	18	12%
No. of Comorbidities *^	1.8	1.5
Range	0–6	
Missing	3	2%
**Follow-up Time in Days**		
Follow-up 1 (~3 months)	107.9	99.5
Range	29–1009	
Follow-up 2 (~12 months)	395.6	217.8
Range	64–1477	
**Baseline Oswestry Disability Index score**	46.7	16.0
Range	11.1–95.6	
Missing	0	0%
	**No.**	**% of total sample**
**Gender**		
Male	65	42%
Female	72	46%
Other	1	1%
Missing	18	12%
**Smoking Status**		
Never smoked/used tobacco	76	49%
Used to smoke/use tobacco	61	39%
Currently smoke/use tobacco	16	10%
Decline to answer	1	1%
Missing	2	1%
**Level of Education**		
Less than high school	7	4%
Graduated from high school or GED	18	12%
Some college or technical school	28	18%
Completed technical school (college)	6	4%
Graduated from college	40	26%
Postgraduate school or degree	42	27%
Decline to answer	1	1%
Missing	14	9%
**Diagnoses ^**		
*Disc Herniation*		
Yes	45	29%
No	102	65%
Missing	9	6%
*Radiculopathy/sciatica*		
Yes	13	8%
No	134	86%
Missing	9	6%
*Spinal stenosis with neurogenic claudication*		
Yes	100	64%
No	47	30%
Missing	9	6%
*Spondylolisthesis (Lytic)*		
Yes	5	3%
No	142	91%
Missing	9	6%
*Spondylolisthesis (degenerative)*		
Yes	34	22%
No	113	72%
Missing	9	6%
**Primary Procedure**		
Lami/disc	98	63%
Instr/fusion alone	20	13%
Instr/fusion w lami disc	31	20%
Missing	7	4%
**Number of Vertebrae Fused if Fusion Surgery**		
Instr/fusion alone		
2	12	8%
3	4	3%
4	3	2%
>4	1	1%
Instr/fusion w lami disc		
2	17	11%
3	8	5%
4	2	1%
>4	4	3%
**Pain Medicine Frequency**		
Not at all	23	15%
Once a week	2	1%
Once every couple days	13	8%
Once or twice a day	54	35%
3 or more times a day	62	40%
Decline to answer	1	1%
Missing	1	1%
**Specific Comorbidities ^** (back-pain excluded)		
Anemia or other blood disorder	5	3%
Arthritis (rheumatoid or unspecified type)	26	17%
Asthma	4	3%
Cancer	7	4%
Depression	13	8%
Diabetes	20	13%
Heart disease	15	10%
High blood pressure	60	38%
Insomnia	5	3%
Kidney disease	1	1%
Liver disease	0	0%
Lung disease	8	5%
Osteoarthritis, degenerative arthritis	49	31%
Stroke	1	1%
Ulcer or stomach disease	6	4%
One or more additional comorbidities	45	29%
Missing	3	2%

† Data reflect baseline values for all variables except follow-up time in days. * The comorbidities included in the survey as potential options changed over time, but generally about a dozen options were included. ^ For these topics, a non-response was counted as the absence of the event in question (e.g., no disease).

**Table 2:** Cross-tabulation of number of patients per group at the two time intervals.

		Groups Classifying ODI Change 2nd Interval (3 mo. to 12 mo.)					
		Disability Worsened	No Effect	Small Effect	Medium Effect	Large Effect	Total
**Groups classifying ODI change 1st Interval** **(pre-surgery to 3 mo.)**	Disability worsened	1	0	2	0	6	9
	No effect	6	1	1	2	0	10
	Small effect	4	4	1	1	4	14
	Medium effect	6	1	2	3	5	17
	Large effect	47	17	18	9	15	106
	Total	64	23	24	15	30	156

**Table 3:** Appraisal change for two time windows: Cohen’s d.

		ODI Change Group Baseline to 3 Months (1st Interval)						ODI Change Group 3 Months to 12 Months (2nd Interval)				
	Appraisal Item	Disability Worsened	No Effect	Small Effect	Medium Effect	Large Effect		Disability Worsened	No Effect	Small Effect	Medium Effect	Large Effect
		n = 9	n = 10	n = 14	n = 17	n = 106		n = 64	n = 23	n = 24	n = 15	n = 30
*Sampling of Experience*	Worst moments	0.544	0.000	−0.491	−0.243	−0.646	Worst moments	0.163	0.418	−0.038	−0.232	−0.183
	Emphasize positive	−0.504	−0.707	0.000	0.346	0.343	Emphasize positive	−0.158	−0.348	−0.086	−0.065	0.321
	Recent few weeks	0.267	−0.316	0.109	0.000	−0.196	Recent few weeks	−0.093	−0.493	−0.158	0.054	−0.420
	Relevant past 3 mo.	0.000	0.000	0.058	0.433	−0.234	Relevant past 3 mo.	−0.329	0.084	−0.346	0.128	−0.077
	Balance positive/negative	−0.185	−0.194	0.539	0.211	0.226	Balance positive/negative	−0.281	−0.254	0.216	0.567	0.194
	Recent flare-ups	0.095	−0.224	0.185	−0.200	−0.457	Recent flare-ups	0.012	−0.327	−0.042	−0.229	−0.025
	Future	0.185	−1.037	−0.584	0.323	0.030	Future	−0.245	−0.228	−0.246	−0.165	−0.216
	Focus on spinal condition	−0.120	−0.523	−0.467	−0.676	−0.805	Focus on spinal condition	0.232	−0.160	−0.357	−0.204	−0.463
	Relationships	−0.142	−0.572	0.185	0.122	−0.180	Relationships	−0.086	−0.240	0.412	0.393	−0.143
	Doctor told	0.312	−0.254	−0.214	−0.081	−0.102	Doctor told	−0.255	−0.184	0.171	0.106	−0.204
	First reaction	0.203	0.349	0.070	−0.069	0.207	First reaction	−0.223	−0.134	−0.379	0.422	0.069
	Not complain	0.000	−0.372	0.000	0.323	0.106	Not complain	−0.065	−0.234	−0.240	−0.056	0.000
	Seriousness	0.438	−0.316	−0.087	0.174	−0.481	Seriousness	0.144	0.041	−0.216	−0.098	−0.052
	Others with same condition	0.360	−0.101	0.185	0.000	0.000	Others with same condition	−0.035	−0.121	−0.057	−0.483	−0.135
*Standards of Comparison*	Healthy others	0.000	−0.396	0.111	−0.368	−0.079	Healthy others	0.024	−0.300	−0.209	−0.166	−0.224
	Doctor said	0.000	0.000	0.098	−0.056	−0.147	Doctor said	−0.281	−0.177	−0.171	−0.338	−0.046
	Perfect health	0.356	−0.176	−0.215	0.283	−0.136	Perfect health	0.081	−0.342	0.250	−0.128	−0.315
	Life working for	0.558	−0.163	0.086	0.093	−0.239	Life working for	0.013	−0.443	−0.254	−0.126	−0.085
	Way others see you	−0.280	−0.218	0.145	−0.112	−0.030	Way others see you	−0.028	−0.223	−0.232	−0.308	−0.218
	People your age	0.000	0.194	0.086	−0.320	−0.227	People your age	0.071	−0.192	0.037	0.050	0.112
	Time before spinal condition	0.272	0.296	−0.145	0.045	−0.357	Time before spinal condition	0.000	−0.188	−0.367	−0.137	−0.200
*Conditional formatting according to published interpretation standards for Cohen’s d: 0.20 to 0.49 is a small effect, 0.50−0.79 is a medium effect, and > 0.80 is a large effect.*												
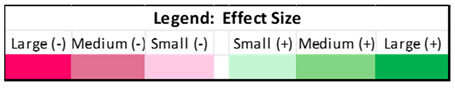												

## Data Availability

The data used in these analyses are confidential and thus not able to be shared.

## Notes

1 Hedge’s g is interpreted as follows: 0.15 to 0.359 is a small effect, 0.36–0.649 is a medium effect, and >0.65 is a large effect.
